# Examining ecotourism intention: The role of tourists' traits and environmental concerns

**DOI:** 10.3389/fpsyg.2022.940116

**Published:** 2022-07-28

**Authors:** Farrukh Rafiq, Mohd Adil, Jei-Zheng Wu

**Affiliations:** ^1^Department of Business Administration, College of Administrative and Financial Sciences, Saudi Electronic University, Jeddah, Saudi Arabia; ^2^Department of Management Studies, National Institute of Technology (NIT) Hamirpur, Hamirpur, India; ^3^Department of Business Administration, Soochow University, Taipei, Taiwan

**Keywords:** extraversion, neuroticism, environmental concern, ecotourism, environmental knowledge, personality traits

## Abstract

The study offers new insights by examining the influence of personality traits (extraversion and neuroticism) on tourists' intentions to visit ecotourism sites using the lens of the theory of planned behavior. It also investigates whether environmental knowledge moderates the effect of extraversion, neuroticism, and environmental concern on tourists' ecotourism intentions. We applied structural equation modeling on 350 responses collected through the Amazon M-Turk platform. Results highlight that extroverts are more likely to express ecotourism intentions than neurotic tourists. However, it was also noted that neurotic tourists' intention to visit ecotourism sites could be influenced if their environmental concerns were emphasized. The study offers important new insights to managers, policy-makers, and practitioners about the roles of personality traits, environmental knowledge, and environmental concern vis-a-vis their relative significance in shaping tourists' decision-making and choices for ecotourism sites. As a result, managers/practitioners need to devise specific communication strategies to enhance awareness and a sense of responsibility among neurotic tourists.

## Introduction

The increasing awareness of environmental issues, limited natural resources, and high environmental costs drive sustainability adoption. Tourism is widely acknowledged as one of the world's largest industries, contributing to sustainability (Sadiq et al., 2022). Therefore, it is possible with the help of a balance between tourism management and the safety of resources: the relationship is known as ecotourism (Lo and Janta, 2020). It has been strongly referred to as a source of rural welfare focusing on endangered species/vulnerable societies (Fennell, 2001). Ecotourism is defined by Allcock and Evans-Smith (1994, p. 15) as “nature-based tourism that includes an educational component and is managed to be sustainable.”

Previously, individuals from developed nations were considered familiar with ecotourism. Nevertheless, third-world nations gradually adopted this trend, which changed how tourists in developing countries viewed environmental safety (Jalani, 2012). As a result, Cabral and Dhar (2019) argue that emerging economies have much potential to contribute to ecotourism. They will attract more visitors to their ecotourism spots in the coming years. Likewise, India has the potential to become one of the leading ecotourism destinations shortly (Sadiq and Adil, 2021), as it has two major bio-diversity zones—the “Western Ghats” and the “North-East Himalayas.” In addition to being a mega-diversity nation, India has over 750 protected areas, including wildlife sanctuaries, conservation areas, community reserves, and national parks (Cabral and Dhar, 2019). These constitute ~5 percent of India's geographical area (Puri et al., 2019). Researchers (Cabral and Dhar, 2019; Puri et al., 2019; Sadiq and Adil, 2021) pointed out that India is still in the embryonic stage in this research area. Due to the relatively new concept of ecotourism in emerging economies, research on Indian tourists' intentions to visit ecotourism destinations would be of interest. Hence, the current study examines Indians' intentions to visit ecotourism sites.

One question, which always remains a pressing issue, is “Why do tourists/consumers behave responsibly or irresponsibly?” Researchers from different academic disciplines have investigated this question. In most studies, emphasis has been placed on understanding the complexity associated with consumers' environmental behavior (Joshi and Rahman, 2015). These include social norms, consumer values, attitudes, perceived behavior control, and trust (White et al., 2019). The literature suggests that researchers are increasingly interested in understanding the drivers of green behavior, but little is known about how negative valence inhibits conservation behavior, e.g., Kaida and Kaida (2017). Therefore, it is pertinent to identify the underlying causes of pro-environmental behavior and its barriers. The present study intends to test a model of “pro-environmental behavior” (ecotourism intention) for extrovert and introvert visitors at an ecotourism site that involves human interaction with the environment (Thompson et al., 2018; Cabral and Dhar, 2019). The study contributes significantly to research on environmental safety (Bertella and Acquarone, 2018).

There is evidence that environmental safety and personality are significantly linked (Kaida and Kaida, 2019; Sadiq, 2019; Sadiq et al., 2021a), so discussing how personality traits impact environmental choices is important. In addition, several scholars have examined the role of personality traits in ethical behavior (Moghavvemi et al., 2017; Sadiq et al., 2021b). Research indicates that individuals have either a pro-environmental or an anti-environmental personality (Brick and Lai, 2018), which results in either pro- or anti-ethical behavior. For example, extraversion describes how an individual is assertive, social, talkative, and outgoing, whereas neuroticism is the opposite (McCrae and Costa, 1985; Busic-Sontic et al., 2017).

Literature suggests that pro-environmental behavior has been measured using a variety of theoretical perspectives, including psychological and personality characteristics (Yadav and Pathak, 2016a; Moghavvemi et al., 2017; Sadiq et al., 2021a). However, the use of personality traits to predict pro-environmental behavior (in this study, it is ecotourism intention) is limited, especially in emerging countries like India. As a result, this presents a research gap. This study fills a gap in the tourism marketing literature by addressing the research gap identified in the existing literature. According to the authors, almost no study has looked at the significance of extraversion and neuroticism in explaining the adoption of ecotourism in developing countries. Furthermore, we used environmental concern (as a mediator) and environmental knowledge (as a moderator) within the framework of the conceptualized model.

## Theoretical framework

Over the last four decades, scholars have extensively investigated the link between consumers' traits, environmental concerns, and pro-environmental behavior/ethical behavior. Research has previously tried explaining the complex factors involved in pro-environmental behavior using different theories, including “the theory of reasoned action” (Ajzen and Fishbein, 1975), “the theory of planned behavior” (TPB) (Ajzen, 1991), “the normative decision-making model” (Schwartz and Howard, 1980), “the norm activation model” (Schwartz, 1968), among others.

Specifically, the TPB has been used extensively to predict consumption (Coşkun et al., 2017). It includes predicting green buying behavior (Paul et al., 2016), green hotel visits (Verma and Chandra, 2018), travelers' online travel purchase behavior (Sadiq et al., 2021d), and recycling behavior (Passafaro et al., 2019). Furthermore, a review of available literature on the theory shows that consumers can execute the given behavior for their reasons and control it; thus, their behavior is planned. In the TPB, situation-specific thinking is recognized as a factor predicting behavior through attitudes, subjective norms, perceived behavior control, and beliefs (Ajzen, 1991).

Researchers have, however, raised concerns about TPB theory for several reasons. For instance, Coşkun et al. (2017) noted that attitude alone is not enough to explain intentions and, therefore, requires additional factors/drivers to explain individiuals' overall behaviors (Sadiq et al., 2021d). Furthermore, Ajzen (1991) posited that the TPB could be further modified/extended by including other variables if such alterations enhance the explanation of intentions and behaviors. More recently, Poškus and Žukauskiene (2017) and Poškus (2020) highlighted that when one examines “introversion-extroversion” or additional dispositional variables in an event to explain behavior, one can gain invaluable insight into behavior by understanding how it affects the behavior of interest (Fishbein and Ajzen, 2010). As a result, previous scholarly articles have shown that the TPB has been extended by adding variables such as environmental concern (Yadav and Pathak, 2016a), environmental knowledge (Yadav and Pathak, 2016a), health concern (Yadav and Pathak, 2016b), and tourist motivation (Hsu and Huang, 2012), to name a few.

**Figure 1:**
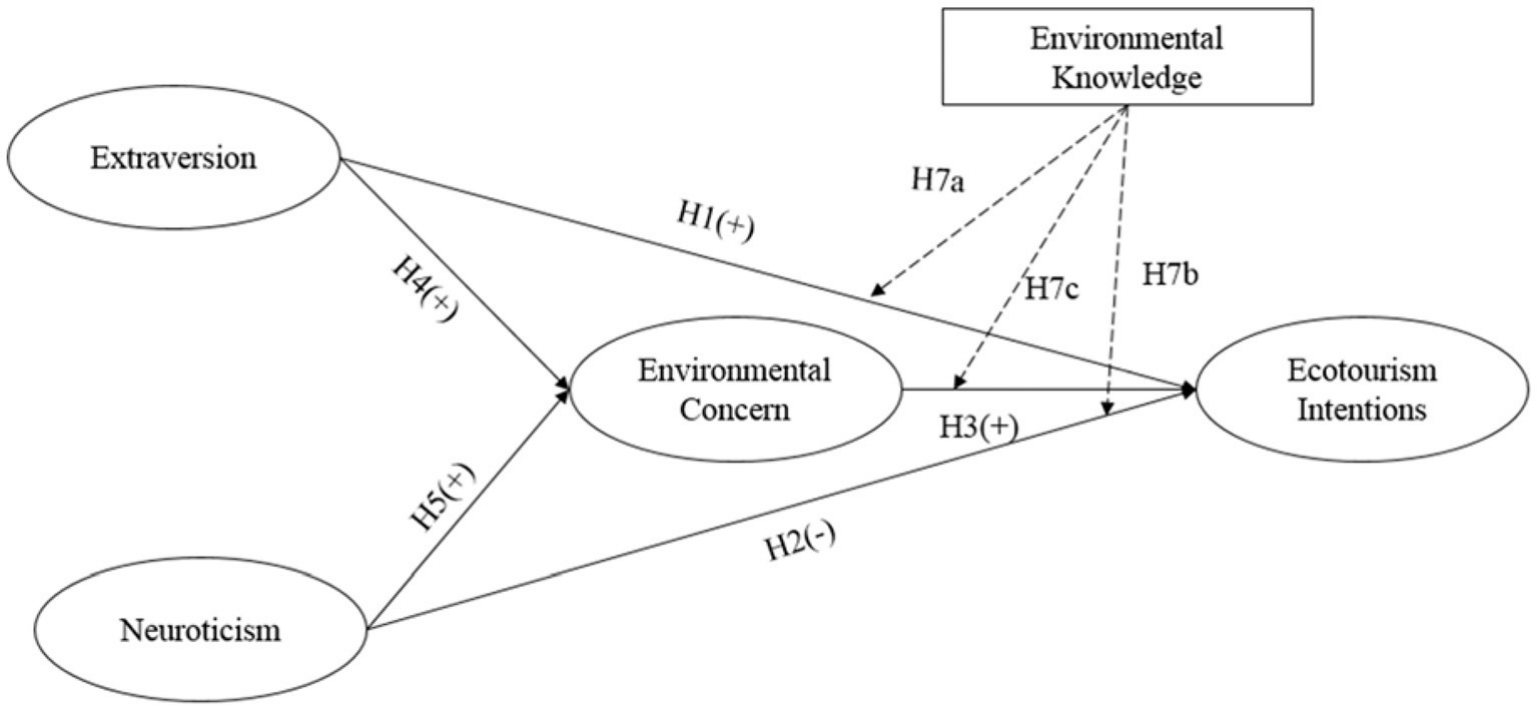
Conceptual model.

Following Bamberg's (2003, p. 21) suggestion, we considered environmental concern as against the “general attitude” toward the environment. Our conceptual model (see Figure 1) tests the interaction between extraversion, neuroticism, environmental concern, and ecotourism intention. Within the conceptual model, we employed environmental concern (Bamberg, who defined the environmental concern as the environmental attitude) as a direct determinant of ecotourism intention and a mediator between extraversion/neuroticism and ecotourism intention (see Figure 1).

### Development of a research model

#### Extraversion and ecotourism intentions

Individuals with extraversion traits are pro-social with positive emotions, free to talk, optimistic in their approach, and energetic (McCrae and Costa, 1985; Busic-Sontic et al., 2017). Coelho et al. (2017) draw on literature demonstrating that extroverts seem more helpful because they foster “the anticipation of positive outcomes such as gratitude rather than the costs of helpful behaviors” and therefore, they contribute to society. In other words, highly extroverted individuals are more likely to engage with the surrounding environment, thus enhancing their contribution “to activities that reduce the negative environmental impacts of mankind on the environment” (Sadiq, 2019). For instance, extroversion motivates individuals to exhibit actions beneficial for communities and the environment, such as adopting renewable energy (Busic-Sontic et al., 2017). Coelho et al. (2017) further argued that a positive outcome broadens the scope of action, encouraging people “to engage with their environments and partake in activities, many of which are adaptive for the individual, its species, or both,” enabling them to prepare for the future. Hence, extroverts are more likely to mix with people, adopt social changes, and have an orientation to achieve social wellbeing (Kirkwood, 2017).

The extant literature offers mixed results about the influence of extraversion on an individual's concern or intention toward the environment. For example, Hirsh (2010) and Hirsh and Dolderman (2007) argued that extraversion does not hold a significant relationship with environmental concern, while, Milfont and Sibley (2012), Busic-Sontic et al. (2017), and Sadiq et al. (2021b) reported contradictory results. However, pro-environmental literature in tourism indicates that extraversion helps develop a tourist's positive intention/behavior. For example, Kvasova (2015) found that extrovert tourists strongly prefer to engage in eco-friendly behavior at their destinations. Similarly, Tang and Lam (2017) and Verma et al. (2017) argued that tourists with high extroversion traits have much stronger intentions to travel to sustainable destinations, including green hotels. Hence, taking cues from previous studies, we hypothesize the following positive association:

*Hypothesis 1 (H1)*: Extraversion is positively associated with ecotourism intentions.

#### Neuroticism and ecotourism intentions

Individuals with high levels of neuroticism lack the resources they require physically, socially, and psychologically (Coelho et al., 2017). Resources must display ecotourism intention, “an indication of an individual's readiness to perform a given behavioral” (Yadav and Pathak, 2016b, p. 123). Furthermore, neuroticism demotivates individuals from participating in social activities due to low self-esteem (Kvasova, 2015). Neurotic individuals are stressed, anxious, fearful, and insecure (McCrae and Costa, 1985; Coelho et al., 2017). Coelho et al. (2017) found that individuals high in negative affect (neuroticism) tend to help others only when the benefits outweigh their costs, such as when people believe they have hurt others or think that helping others will alleviate their own misery. It may be noted that since pro-environmental behavior incurs “short-term costs,” individual weighs the costs and benefits of pro-environmental behavior before making any decision. Hence, individuals with low psychological resources are less inclined to exhibit pro-environmental behavior (Coelho et al., 2017; Sadiq et al., 2021b). Notably, the existing pro-environmental literature in the tourism context offers mixed evidence about how neuroticism influences tourists' concerns or intentions toward the environment. For example, Kvasova (2015) and Verma et al. (2017) found that neuroticism is significantly and positively associated with tourists' pro-environmental behavior/intention, while Sachdeva and Hafiz (2019) reported contradictory results. However, drawing on research such as Milfont and Sibley (2012), Poškus (2018), and Sadiq et al. (2021b) that indicate pessimism/neuroticism significantly and negatively influences an individual's environmental orientation/engagement at the country level, we propose the following negative relationship:

*Hypothesis 2 (H2):* Neuroticism is negatively associated with ecotourism intentions.

#### Environmental concern and ecotourism intentions

Environmental concern refers to “the degree to which people are aware of problems regarding the environment and support efforts to solve them or indicate the willingness to contribute personally to their solution” (Dunlap and Jones, 2002, p. 482). Individuals are becoming more eco-conscious, which leads to more pro-environmental behavior (Dhir et al., 2021a; Sadiq et al., 2021c). Furthermore, environmental concern positively influences ecotourism intentions (Rhead et al., 2015; Sadiq et al., 2021a). On the other hand, Yadav and Pathak (2016a) claimed that environmental concern is weakly associated with individuals' intentions, while a few scholars observed a strong relationship (Huang and Liu, 2017; Kumar et al., 2021). Despite the varied viewpoints in the literature, this research hypothesizes the relationship as follows:

*Hypothesis 3 (H3)*: Environmental concern is positively associated with ecotourism intentions.

#### The indirect effect of environmental concern

Individuals with an extroversion trait are likely to focus on negative information that is important to them [such as information related to coronavirus] (Moradi et al., 2019; Chen et al., 2020). Further, extraversion significantly influences individuals' cognitive ability, i.e., thought processes that improve their problem-solving ability. In contrast, introverted (high on neuroticism) individuals tend to be low cognition. Therefore, they focus only on the negative side of any information that leads them to lose their psychological resources (Coelho et al., 2017; Moradi et al., 2019). Extrovert individuals are believed to recall positive and relevant negative information for future use, motivating them to think positively (Busic-Sontic et al., 2017).

On the contrary, introverted individuals are considered to remember the negative information for future use (Pang et al., 2019), which motivates them to think negatively. Extant literature suggests that introverted individuals focus on ‘negative environmental information,' thus developing a positive environmental concern. Furthermore, extrovert individuals exhibit orientation toward understanding the environment-related issue, resulting in a positive environmental concern. Thus, we hypothesize:

*Hypothesis 4 (H4)*: Extroversion is positively associated with environmental concern.
*Hypothesis 5 (H5)*: Neuroticism is positively associated with environmental concern.

Prior relevant literature supports the claim that environmental concerns are associated with ecotourism (Yadav and Pathak, 2016a,b). Further, a few studies focused on mediating the role of environmental concern between positive or negative personality characteristics and pro-environmental behavior. For instance, environmental concern has been tested to mediate extroversion/neuroticism and pro-environmental behavior. Similarly, the mediating role of environmental concern between effect (positive/negative) characteristics and pro-environmental behavior was studied (Coelho et al., 2017). Therefore, this study hypothesized as follows:

*Hypothesis 6.1 (H6.1)*: The link between extraversion and ecotourism intention is mediated by environmental concern.
*Hypothesis 6.2 (H6.2)*: The link between neuroticism and ecotourism intention is mediated by environmental concern.

#### The moderating role of environmental knowledge

Environmental knowledge refers to “a general knowledge of facts, concepts, and relationships concerning the natural environment and its major ecosystems” (Fryxell and Lo, 2003, p. 45). Gauging individuals' familiarity with the environment is essential to understanding any nation's eco-friendly movement (Geiger et al., 2019). Several scholars argued that environmental knowledge is important in determining an individual's eco-friendly decision capability (Cheung and Fok, 2014; Dhir et al., 2021a). Further, scholars such as Taufique et al. (2017) and Fang et al. (2018) studied environmental knowledge as an important predictor of environmental-friendly behavior. At the same time, a few (Suki and Suki, 2015; Kumar et al., 2017; Sadiq et al., 2021b) have treated environmental knowledge as a moderator between the association of environmental-friendly behavior with its drivers. Notably, Suki and Suki (2015) noted that individuals with high environmental knowledge showed greater associations between their attitudes [environmental concern in this research; Bamberg (2003) describes it as a “general attitude” toward the environment] and intentions to adopt pro-environmental behavior. In addition, Sadiq (2019) and Sadiq et al. (2021b) state that individuals having extrovert characteristics tend to have a deep understanding of environmental conditions.

In contrast, individuals with neurotic characteristics are more likely to avoid news related to environmental degradation, resulting in low environmental knowledge. Thus, extroverts tend to adopt eco-friendly behavior as they focus on positive information related to the environment; however, introverts (high on neuroticism) are more likely to avoid environmental-friendly behavior due to low awareness or only focus on negative information related to the environment. Therefore, this study assumes that if tourists are offered a proper environment wherein messages to enhance environmental awareness are communicated, it is more likely that extrovert and introvert tourists show strong intention to adopt environmental-friendly behavior and help bridge the “concern-behavior” gap. Hence, we hypothesized:

*Hypotheses 7a-c (H7a-c):* Environmental knowledge (high vs. low) significantly moderates the association of ecotourism intentions with extraversion, neuroticism, and environmental concern.

## Methodology

### Questionnaire development

This study has divided the questionnaire into a) socio-demographic questions and (b) questions related to adopted variables in this study's research model. In line with Kvasova (2015), the 8-item of extraversion and neuroticism was adopted from Donnellan et al. (2006)'s “mini International Personality Item Pool—Five-Factor Model” 20-item scale. See Appendix 1 for items related to the variables. Some example items for extraversion are: “Am the life of the party” and “Don't talk a lot”; similarly, example items of neuroticism are: “Have frequent mood swings” and “I am relaxed most of the time.” In addition, 5-point Likert scales were used to rate each personality trait, anchored as “1 = Very Inaccurate and 5 Very Accurate.” Ecotourism intentions were measured through a 3-item scale adapted from Pham and Khanh (2021). Example items of ecotourism intention are: “I will choose ecotourism in my traveling,” and “I intend to visit an ecotourism destination within a foreseeable future.” Environmental concern was measured through a 4-item scale by Sadiq et al. (2021a). Some environmental concern items are, “The balance of nature is very delicate and can be easily upset,” and “Humans must maintain the balance with nature to survive.” Finally, the level of environmental knowledge was assessed using a 3-item scale drawn from Kumar et al. (2017). Sample items are: “Ecotourism is a primary way to reduce pollution,” and “Ecotourism is a substantial way to reduce wasteful use of natural resources.” The items related to environmental concern, environmental knowledge, and ecotourism intention were captured using the 5-point Likert scale, anchored as “1 = Strongly Disagree and 5 = Strongly Agree.”

### Selection of respondents

We used a multivariate analysis technique, where the researcher needs a sample size of 10–15 times the number of questions asked to measure the employed variables (Hair et al., 2014). Since this study uses 18 questions to measure the five variables, the sample size threshold should be 18^*^15 = 270. Furthermore, ecotourism literature suggests that a sample size of 200–400 provides reliable results (Hussain et al., 2015; Sadiq and Adil, 2021). As a result, 350 respondents were surveyed, which is greater than the threshold level under the current study, i.e., 270.

Given COVID-19 and its related protocols, the authors decided to collect the data from the well-established Amazon M-Turk platform. This platform often provides reliable and conclusive findings (Sadiq et al., 2022). Further, inclusion criteria have been set up in order to avoid possible biases in the data, i.e., (a) the respondents must have an acceptance rate of 90% or more on the M-Turk, (b) the respondents must be of 18 years or old as phenomena associated with the ecotourism is difficult for adolescents to understand (Sadiq and Adil, 2021); (c) respondents should be a resident of India; (d) respondents should have visited ecotourism destinations at least once in the last 6 months.

The survey was uploaded on the M-Turk on 28th March 2022 and paused after reaching 350 responses. Of these, 58% (203) were male, while 42% (147) were female, with a median age of 34.7. Most respondents were married (67.4%), held a bachelor's educational degree (82%), and belonged to the Indian Rupees 70,001–90,000 monthly income group. Finally, 39.14% of respondents reported being neurotic, while almost 60% were extroverts.

## Analysis and findings

Two-step “structural equation modeling” (SEM) tested the research model using the AMOS software. Then, following (Dhir et al., 2021a,b), a macro process test was used to assess mediation and moderation.

### Descriptive analysis

To explore missing data, the current study used the test of frequency. According to the results, there are no missing values in the dataset. As a second step, we used “Cook's distance” to ensure the data does not contain any abnormal responses (i.e., outliers). In cases where the response distance is ≥1, it is recommended to consider that particular observation as an outlier and exclude it from the analysis. Results clearly show that none of the responses had Cook's distance values above 1, so there were no abnormal or unusual responses (Sadiq et al., 2021a). In addition, researchers (Gupta and Adil, 2014; Ullah and Adil, 2016) suggest that before conducting advanced statistical analysis on the data, assumptions related to the normality of the data should be determined. As a result, we conducted preliminary statistical tests (i.e., skewness and kurtosis). The outcome indicates that all values fall within the recommended threshold level of +3 and −3 (George and Mallery, 2018), indicating that the data has no abnormality issues. Finally, we also examine the issue of “common method bias” (CMB). We conducted a “Harman's single factor test” to test CMB, resulting in a single factor explaining 38.3% of the variance, which is within the 50% limit. Therefore, the data were free of CMB.

### Confirmatory factor analysis

As part of our research, a CFA was performed to assess the validity and reliability of our model. As a first step, we checked the model indices (CMIN/df = 1.87; CFI = 0.95; TLI = 0.96; RMSEA = 0.047), which were satisfactory. In addition, the composite reliability and Cronbach's alpha were investigated to establish the reliability of constructs; both values were above the threshold, confirming their reliability. Further, we checked the “average variance extracted” (AVE) to assess the convergent validity. Each variable had an AVE > 0.5 (see Table 1), indicating convergent validity. The AVE values of the square root of intercorrelation between all constructs were compared to assess discriminant validity. According to the results, the value of each construct's variable exceeds the inter-correlation value, establishing discriminant validity (see Table 2).

**Table 1:** Reliability and convergent validity.

Item	Item code	Factor loading	AVE	CR	α
Extraversion	E1	0.74	0.60	0.86	0.85
	E2	0.80			
	E3	0.83			
	E4	0.73			
Neuroticism	N1	0.78	0.59	0.85	0.83
	N2	0.76			
	N3	0.79			
	N4	0.74			
Environmental concern	EC1	0.87	0.65	0.88	0.86
	EC2	0.79			
	EC3	0.83			
	EC4	0.75			
Ecotourism intention	EI1	0.72	0.63	0.83	0.82
	EI2	0.82			
	EI3	0.85			

AVE, Average variance extracted; CR, Composite reliability; α, Cronbach's Alpha.

**Table 2:** Discriminant validity.

	Mean	SD	E	N	EC	EI
E	4.226	1.39	0.60			
N	3.218	0.79	−0.41	0.59		
EC	3.629	1.03	0.53	0.57	0.65	
EI	4.187	1.32	0.56	−0.42	0.51	0.63

SD, Standard Deviation; E, Extraversion; N, Neuroticism; EC, Environmental Concern; EI, Ecotourism Intention.

### Path analysis

As a result of path analysis, extraversion on ecotourism intention (H1: β = 0.534^***^) and extraversion on EC (H4: β = 0.422^***^) were significant and positive. Therefore, H1 and H4 were supported (see Table 3). Similarly, the influence of neuroticism on ecotourism intention (H2: β = −0.147^*^) and EC (H5: β = 0.591^***^) were significant. Therefore, both hypotheses (H2 and H5) were supported. After that, we check the variance explained in EC and ecotourism intention. The results indicate that the variance explained in EC was 44.74%, and ecotourism intention was 43.10%.

**Table 3:** Results of path analysis.

Hypothesis	Estimate	***P***	T	Result of hypothesis
E→EI (H1)	0.534	<0.001	12.999	✓
N→EI (H2)	−0.147	<0.05	−3.126	✓
EC→EI (H3)	0.165	<0.001	3.481	✓
E→EC (H4)	0.422	<0.001	11.938	✓
N→EC (H5)	0.591	<0.001	15.889	✓
***R^**2**^***-values for EC = 0.4474 & PEI = 0.4310				
Model Indices: CMIN/DF = 1.91; CFI = 0.94; TLI = 0.95; RMSEA = 0.051				

(✓) Hypothesis supported; E, Extraversion; N, Neuroticism; EC, Environmental Concern; EI, Ecotourism Intention.

### Indirect effect

Regarding the indirect effects of EC, we proposed two hypotheses (H6.1 and H6.2). We used Model 4 of the SPSS software to measure the indirect effects. The H6.1 was only partially accepted because extraversion continues to significantly affect ecotourism intentions even when an EC factor is present (see Table 4). In addition, H6.2 was partially accepted as the “direct effect” of neuroticism on ecotourism intention and remains significant in the context of environmental concern.

**Table 4:** Indirect effect.

Hypothesis number	Path	Indirect effect	Type of Mediation	LLCI	ULCI	Supported?
H6.1	E→ EC→ EI	0.069	*Partial mediation*	0.024	0.114	✓
H6.2	N→ EC→ EI	0.098	*Partial mediation*	0.035	0.172	✓

### Moderation effect

**Figure 2:**
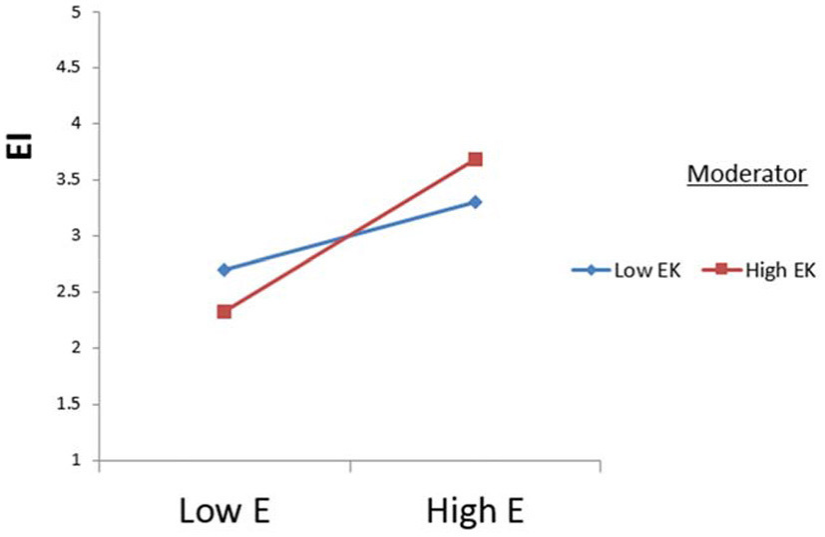
Moderating effect of environmental knowledge (EK) on the relationship between extraversion (E) and ecotourism intention (EI).

**Figure 3:**
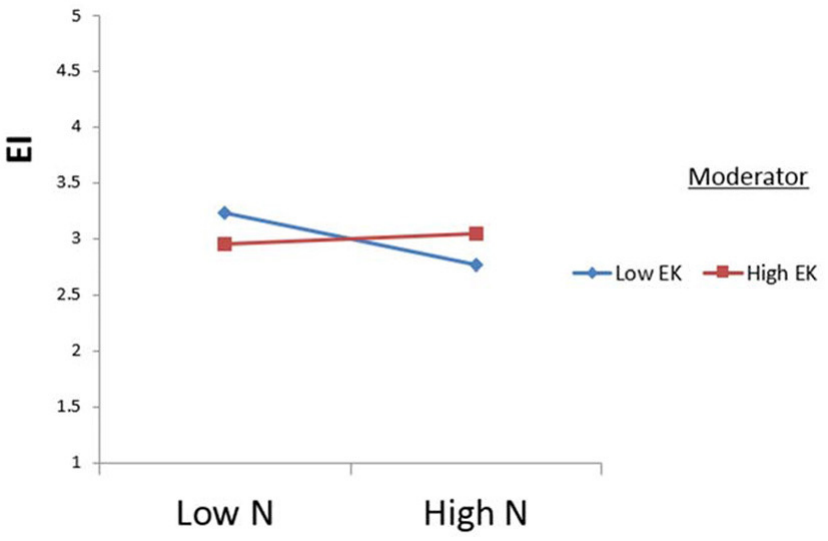
Moderating effect of environmental knowledge (EK) on the relationship between neuroticism (N) and ecotourism intention (EI).

**Figure 4:**
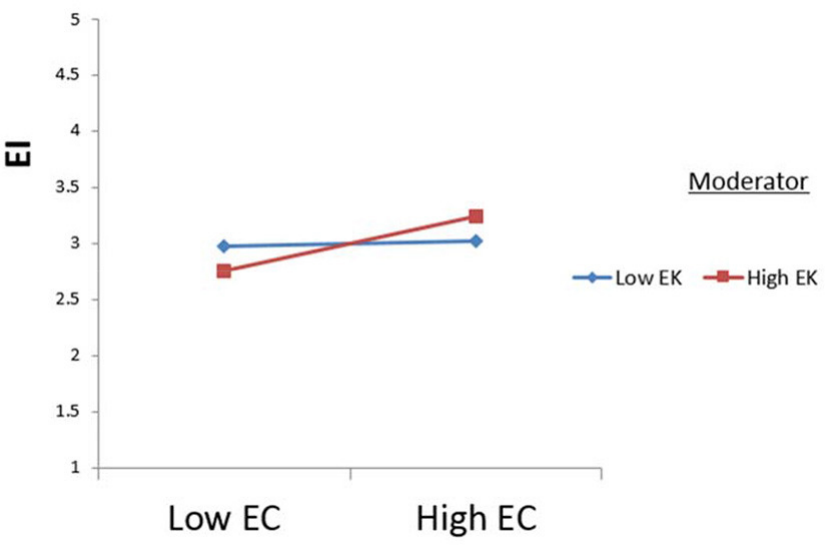
Moderating effect of environmental knowledge (EK) on the relationship between environmental concern (EC) and ecotourism intention (EI).

The study tested the moderating effects of environmental knowledge using Process Macros (model 1). The results indicate that extraversion is significantly moderated by environmental knowledge concerning ecotourism intention (see Figure 2). Additionally, the relationship between neuroticism and ecotourism intention was significantly moderated by H7b (see Figure 3). Therefore, H7c was supported (see Figure 4).

## Discussion

The current study focuses on understanding the significance of personality traits in motivating tourists to take up ecotourism. Besides, it aims at bridging the concern-behavior gap. Therefore, we tested five direct, two indirect, and three moderating hypotheses to test our research model. This study's finding supports H1 and H2, wherein extraversion and neuroticism are positively and negatively associated with ecotourism intention. The current study highlights that extrovert tourists would conduct eco-friendly behavior by visiting ecotourism sites. This study's results concur with Kvasova (2015) and Busic-Sontic et al. (2017). There could be a justification for this by arguing that extrovert tourists can better deal with difficult situations, such as being willing to spend more money at ecotourism sites to minimize their environmental impacts (Kesenheimer and Greitemeyer, 2021).

Meanwhile, the finding related to H2 indicates that neuroticism negatively impacts ecotourism intentions. Although this finding is in line with Poškus and Žukauskiene (2017), the result in the present study supports that introvert (high in neuroticism) ecotourism visitors tend not to display intentions to visit ecotourism destinations. Instead, introvert tourists display withdrawal behavior (Guo et al., 2018) since such tourists are low on their psychological resources, such as coping capabilities. Therefore, introverted tourists tend to exhibit negative ecotourism intentions due to their proneness to stress and withdrawal behavior under difficult situations such as cleaning the sea to clean the environment.

Considering one of the core assumptions of the TPB that “behavioral intentions are influenced by the attitude” (LaMorte, 2019), we proposed H3. Since researchers such as Bamberg (2003) and Sadiq (2019) suggest that environmental concern can be viewed as a general attitude toward the environment, we used environmental concern rather than a general attitude. Our study supports H3, which is also in line with Huang and Liu (2017), and Pham and Khanh (2021). Further, the result indicates that the environmental concern of an Indian ecotourism tourist converts into ecotourism intention with a weak association, which results in a concern-behavior gap. The possible justification is that India is an emerging nation with a high collectivist orientation (Sadiq et al., 2020). Hence, there is a hierarchical system imposed within each society, and an individual has to follow the hierarchy system. For example, a visitor wishes to do some charity to local communities at the ecotourism sites for their welfare; however, one has to consult the social group head before doing so. Therefore, this possibly leads to widening the concern-behavior gap.

The relationship between the extraversion/neuroticism and environmental concern is positive and significant; having a positive confidence interval with no zero indicates that H4 and H5 were supported. The current research highlights that extravert tourists have an environmental concern. It implies that extrovert tourists look at environmental problems through a positive lens to resolve environmental degradation (Busic-Sontic et al., 2017). Further, our study found that introverted tourists have strong environmental concerns. The possible justification is that introvert tourists are low on psychological resources (Sadiq et al., 2021b); therefore, they tend to focus on negative information related to any issues such as degradation of the environment. Thus, this possibly results in making them environmentally conscious.

In addition, the mediation analysis examined the role of environmental concerns as mediators. A mediation analysis explains that ecotourism intention is indirectly affected by extraversion/neuroticism through environmental concern, which partially supports H6.1 and H6.2. These findings concur with Sadiq et al. (2020), who found optimism/pessimism [having similar characteristics as extraversion/neuroticism (Sadiq, 2019)] has an indirect effect on sustainable behavior through the environmental concern. However, our findings contradict from Busic-Sontic et al. (2017). The possible reason for such contradictory results is the study context. Paul et al. (2016) argued that “Indians are more environmentally conscious” and exhibit a low concern-behavior gap (Uddin and Khan, 2016). Besides, Indian tourists are also high on religious values, as indicated in Adil (2021), possibly motivating tourists to translate their environmental concerns into ecotourism intentions.

Additionally, we investigate the effects of environmental knowledge as a moderator (H7a–c). This study found support for all the moderation hypotheses. According to H7a(H7b), environmental knowledge moderates the link between extroverts (introverts) and ecotourism intentions. As Sadiq et al. (2021b) indicate, extroverts (introverts) focus on positive (negative) information; hence, educating tourists about the environment will encourage them (extrovert/introverts) to visit ecotourism sites. In addition, the current research supports H7c, which states that tourists having good knowledge of the environmental quality does significantly influence the translation of concern into ecotourism intention. The result of H7c concurs with Suki and Suki (2015), wherein they found that high knowledge bridges the concern-behavior gap. It may be because tourists high on environmental knowledge tend to significantly translate their concern for the environment into intentions to visit ecotourism destinations; as Ajzen (1991) demonstrated in his proposal on TPB that knowledge plays an important role in the development of an individual's attitude and intention to carry out actions that help to achieve the given goal, thus, the findings of H7c support TPB's key assumption.

### Theoretical and practical implications

Our study offers significant theoretical implications contributing to ecotourism literature and personality traits. First, this study examines factors influencing tourists' intentions to visit ecotourism sites in the context of India. This study found that personality traits are relevant extensions to the TPB, providing a further theoretical contribution. Second, findings suggest that environmental knowledge is the most significant variable influencing the link between environmental concern and ecotourism intention; however, previous studies have ignored this factor in the ecotourism literature. Third, this research provides insight to academicians by integrating environmental concern as a mediator between ecotourism intention and its drivers. Fourth, this research contributes to developing theories around tourists' personalities and conservation behavior. It can be evident from the finding that neurotic tourists can start exhibiting intention to visit ecotourism sites by inducing environmental concern in them, which leads to bridging the concern-behavior gap. Lastly, this is one of the few studies investigating the role of personality traits (extraversion and neuroticism) on ecotourism intention.

Moreover, the results may also be useful to marketers and policy-makers in designing specific strategies and personalized messages to conserve the environment. For example, neurotic tourists are more likely to experience stress when engaging in risky activities (i.e., financial risk). Since the visit to an ecotourism site involves higher costs than conventional tourism, it places a person at financial risk and helplessness (Landry et al., 2018); therefore, neurotic tourists would decide not to visit the ecotourism sites. Similar observations were also made by Liu et al. (2021). They showed that people with higher neurotic traits are more likely to exhibit risk-averse when comparing themselves to others with inferior characteristics.

Similarly, Oehler and Wedlich (2018) found that extroverted individuals are less risk-averse while neurotic individuals are more risk-averse. In the same vein, Oehler et al. (2018) suggest that people who are more neurotic are less likely to hold risky assets in their portfolios. Thus, managers and governments may design marketing strategies to offer financial assistance or subsidies to tourists who perform or intend to visit ecotourism destinations. As a result, it eventually may motivate neurotic tourists to visit ecotourism destinations.

In order to position their product (eco-tourism site), marketers should consider the wellbeing of the destination's environment. Consequently, Indian ecotourists might adopt eco-friendly behaviors since they tend to be influenced and become more familiar with the benefits of the products (Kumar and Kaushik, 2018). Furthermore, marketers can use advertising to communicate negative messages such as “what will happen to the environment if ecotourism does not become popular and is not adopted” to neurotic tourists to induce their environmental concerns and reduce the concern–behavior gap. Moreover, through promotional activities, marketers can make tourists aware of the benefits of ecotourism over conventional tourism. In addition, practitioners could also focus on developing communication strategies and personalized promotional messages to evoke tourists' environmental awareness and the overall effectiveness of their memorable experiences.

## Conclusion

Since personality traits' role in ecotourism has rarely been studied, this study tested a unique research model. This study integrates extraversion and neuroticism with environmental concern and ecotourism intention to examine personality characteristics in adopting ecotourism. Additionally, our study addresses one of the major issues in eco-friendly tourism, such as the concern-behavior gap. It introduced environmental concern as a mediator between personality traits (extraversion/neuroticism) and ecotourism intention. Introducing environmental concerns to neurotic tourists indicates that they will visit ecotourism.

Additionally, we examined environmental knowledge as a moderating variable on the links between ecotourism intention and extraversion, neuroticism, and environmental concern, respectively. Across all tested paths, environmental knowledge showed a significant moderating effect. Consequently, it also reduces tourists' concern-behavior gaps. Therefore, this study enhanced academicians' and managers' understanding of how extroverts and introverts perceive ecotourism.

Like other social science studies, this research also has a few limitations: (1) this study was conducted in a developing nation, i.e., India, which has cultural characteristics different from other developed nations. As cultural factors play a key role in tourists' decision-making, the researchers suggest applying and extending the findings with caution to developed nations. Furthermore, the research model should be tested for robustness in developed nations. (2) We used a cross-sectional survey method in our study, limiting the generalizability of our research's findings as tourists' behavior tends to change with time. Therefore, future researchers are suggested to carry out longitudinal survey studies. (3) the current research requested respondents to complete the self-administrated survey, though we have taken precautions to avoid CMB. Nevertheless, experiments should be conducted to understand the causality between the employed variables better. (4) Further, future researchers can also test the moderating effect of situational factors and visiting experience in further understanding personality characteristics. They may bridge the concern-behavior gap in the ecotourism domain.

## Data availability statement

The datasets presented in this article are not readily available because the datasets analyzed during the current study are not publicly available due to confidentiality and privacy issues. However, could be available on request from the second author. Requests to access the datasets should be directed to MA, adil.dms@nith.ac.in.

## Author contributions

FR: conceptualization and writing. MA: data collection, data analysis, visualization, and writing. J-ZW: project administration and writing and editing. All authors contributed to the article and approved the submitted version.

## Funding

This work was supported by the Ministry of Science and Technology, Taiwan (MOST110-2628-E-031-001).

## Conflict of interest

The authors declare that the research was conducted in the absence of any commercial or financial relationships that could be construed as a potential conflict of interest.

## Publisher's note

All claims expressed in this article are solely those of the authors and do not necessarily represent those of their affiliated organizations, or those of the publisher, the editors and the reviewers. Any product that may be evaluated in this article, or claim that may be made by its manufacturer, is not guaranteed or endorsed by the publisher.

## Appendix 1

**Table:** Measurement scale.

**Variable name (source)**	**Items**
Extraversion (Kvasova, 2015)	Am the life of the party
	Don't talk a lot
	Talk to a lot of different people at parties
	Keep in the background
Neuroticism (Kvasova, 2015)	Have frequent mood swings
	Am relaxed most of the time
	Get upset early
	Seldom feel blue
Environmental Concern (Sadiq et al., 2021a)	The balance of nature is very delicate and can be easily upset
	Human beings are severely abusing the environment
	Humans must maintain the balance with nature in order to survive.
	Human interferences with nature often produce disastrous consequences
Environmental Knowledge (Kumar et al., 2017)	Ecotourism is a substantial way to reduce wasteful use of natural resources
	Ecotourism is a great way to conserve natural resource
	Ecotourism is a primary way to reduce pollution
Ecotourism Intention (Pham and Khanh, 2021)	I will choose ecotourism in my traveling
	I intend to visit an ecotourism destination within a foreseeable future
	I properly choose ecotourism tour
	I think the ecotourism is right
